# Preparation of Lyocell Fibers from Solutions of Miscanthus Cellulose

**DOI:** 10.3390/polym16202915

**Published:** 2024-10-16

**Authors:** Igor S. Makarov, Vera V. Budaeva, Yulia A. Gismatulina, Ekaterina I. Kashcheyeva, Vladimir N. Zolotukhin, Polina A. Gorbatova, Gennady V. Sakovich, Markel I. Vinogradov, Ekaterina E. Palchikova, Ivan S. Levin, Mikhail V. Azanov

**Affiliations:** 1 A.V. Topchiev Institute of Petrochemical Synthesis, Russian Academy of Sciences, 29 Leninsky Prospect, 119991 Moscow, Russia; m.i.vinogradov1989@yandex.ru (M.I.V.); shatokhina@ips.ac.ru (E.E.P.); levin@ips.ac.ru (I.S.L.); 2 Institute for Problems of Chemical and Energetic Technologies, Siberian Branch of the Russian Academy of Sciences (IPCET SB RAS), 659322 Biysk, Russia; julja.gismatulina@rambler.ru (Y.A.G.); makarova@ipcet.ru (E.I.K.); admin@ipcet.ru (V.N.Z.); 1402plngorbatova@mail.ru (P.A.G.); ipcet@mail.ru (G.V.S.); 3 LLC “NTC Biotechcomposite-Dulevo”, Lenina Street 15/1, 142670 Likino-Dulovo, Russia; biotekhkompozit@bk.ru

**Keywords:** miscanthus, miscanthus giganteus, cellulose, constituent composition, N-methylmorpholine-N-oxide, rheology, fiber spinning, lyocell

## Abstract

Both annual (cotton, flax, hemp, etc.) and perennial (trees and grasses) plants can serve as a source of cellulose for fiber production. In recent years, the perennial herbaceous plant miscanthus has attracted particular interest as a popular industrial plant with enormous potential. This industrial crop, which contains up to 57% cellulose, serves as a raw material in the chemical and biotechnology sectors. This study proposes for the first time the utilization of miscanthus, namely Miscanthus Giganteus “KAMIS”, to generate spinning solutions in N-methylmorpholine-N-oxide. Miscanthus cellulose’s properties were identified using standard methods for determining the constituent composition, including also IR and atomic emission spectroscopy. The dry-jet wet method was used to make fibers from cellulose solutions with an appropriate viscosity/elasticity ratio. The structural characteristics of the fibers were studied using IR and scanning electron microscopy, as well as via X-ray structural analysis. The mechanical and thermal properties of the novel type of hydrated cellulose fibers demonstrated the possibility of producing high-quality fibers from miscanthus.

## 1. Introduction

The perennial herbaceous plant Miscanthus has a large growth area and a strong ability to fix CO_2_, lowering its concentration in the atmosphere [1,2]. Although it belongs to the modest winter-hardy plant family, it is most common in locations with a warm and humid environment (East and Southeast Asia), including China, Japan, and Pacific island states [3,4,5]. An analysis of published Miscanthus processing methods enables us to validate a wide range of products, including cellulose and its derivatives as well as high-tech biosynthesis products [6,7,8,9]. In European countries, an international scientific program for Miscanthus cultivation and processing has been implemented [10,11]. The Russian Miscanthus varieties include *Miscanthus sacchariflorus* “Soranovsky” [12], Miscanthus giganteus “KAMIS” and “FORTIS” [13]. Miscanthus “KAMIS” is a tall upright bush (up to 3 m) that does not produce seeds. Miscanthus reproduces using rhizomes. The average yield of green mass is around 12.8 t/ha. The vegetative season is approximately 190 days. Unlike traditional annual crops such as flax and hemp, miscanthus productivity begins in the second or third year and can extend for up to 20 years.

Miscanthus has a rather different constituent ingredient composition to other perennial and annual sources of cellulose. The cellulose content in miscanthus can reach 57% [2,14,15]. In contrast, its percentage in hemp fibers is 91%, in flax it is 92%, and in cotton it is 97%, and its percentage in raw wood materials can reach 50% [16,17,18,19]. Found in miscanthus in roughly 13–24% of the material [15], lignin is one of the key elements; this proportion is 40–60% greater than in flax and hemp fiber [20,21] and far less than in wood (30%) [22].

Cellulose is a biopolymer with a constantly renewable raw material base. Cellulose is essentially a linear polymer consisting of β-glucose residues linked by β (1→4) glycosidic bonds [23]. Semi-rigid-chain cellulose macromolecules form a complex supramolecular structure due to numerous intermolecular and intramolecular hydrogen bonds [24]. As a result, it cannot be melted or dissolved in many common solvents [25]. Over the last century, various methods for generating cellulose solutions have been developed, including orthophosphoric acid [26], aqueous NaOH solutions [27,28], DMAc/LiCl [29], aqueous zinc chloride solutions [30], N_2_O_4_/DMF [31,32], and ionic liquids [33,34]. However, all of these solvents have been found to have drawbacks that limit their industrial utilization, such as toxicity, high cost, instability of solutions, volatility, and difficulties in regeneration.

In industry, the only method commonly utilized to obtain fibers (films) is the viscose process. This process consists of many complex operations, each of which may consist of several stages. First, cellulose is crushed and treated with a concentrated aqueous alkali solution (mercerization stage), and the resulting system is conditioned for a set period of time to lower the degree of cellulose polymerization. In the following step, alkali cellulose is treated with carbon disulfide (CS_2_) to produce cellulose xanthate. The resultant system dissolves readily in dilute alkali. Fibers are generated in acid baths after the cellulose xanthate has been completely dissolved, filtered, and degassed from the spinning solution. For a more complete description of the viscose process, consider [35,36].

The main drawbacks of the viscose process are the large number of energy-intensive stages and the use of environmentally hazardous non-recyclable substances, which result in a large amount of volatile, solid, and liquid waste (CS_2_, CO_2_, H_2_S, acids, alkali, etc.) being released into the atmosphere; the total amount of waste per ton of the resulting product reaches several tons [37,38,39]. Environmental regulations placed on viscose manufacture in a number of countries have resulted in its relocation to the Asia region, where emission controls are more lenient or completely absent. To date, underdeveloped environmental controls and low electricity and labor costs have enabled the production of low-cost viscose fibers. The ongoing demand for such fibers is driven by rising per capita consumption and global population expansion. To meet the growing demand for hydrated cellulose fibers, a variety of concerns must be addressed, including boosting the supply of soluble cellulose, carbon disulfide, and alkali. According to [40], soluble cellulose must meet a variety of requirements, including a suggested moisture level of no more than 10%, an alpha fraction content of at least 90%, a degree of polymerization of at least 500, and an iron percentage of no more than 8 ppm. Soluble cellulose can be obtained from both perennial and annual plants [41,42]. Alkali is primarily a waste product in the process of producing chlorine from salt, and its production cannot be expanded much without increasing chlorine consumption. High demand for alkali in cellulose separation, spinning solutions, final textile processing, and so on drives up NaOH costs, increasing the risk of viscose production.

The MMO process, which uses N-methylmorpholine-N-oxide (NMMO) as a direct solvent, is an alternative to the viscose method for fiber production [43,44,45,46]. NMMO, as a hygroscopic solvent, can exist in a variety of hydrated forms. With a water concentration of around 13.3% (monohydrate form), NMMO can form crystal hydrates with a melting point of T_m_ ~ 76 °C [47]. The water in NMMO monohydrate does not interfere with the interaction with cellulose’s hydroxyl groups, allowing for a solution with a polymer content (degree of polymerization (DP) < 600) of up to 14%. Reducing the water concentration in NMMO to 10% (T_m_ ~ 120 °C) enhances the solvent’s dissolving capacity, resulting in solutions with cellulose content of up to 18–25%.

Miscanthus has gained popularity in recent years as a constantly renewed source of biomass from which to produce not just cellulose and its derivatives [48], but also glucose solutions, bioethanol, bacterial nanocellulose, and other products [49,50,51,52,53,54]. The issue of processing miscanthus cellulose into fibers is under active development; however, the focus is mostly on creating spinning solutions from refined cellulose, which has properties similar to soluble cellulose [40,55]. The usage of cellulose with partially removed lignin, hemicelluloses, and so on is particularly intriguing.

A review of the literature on miscanthus processing reveals that despite the various ways for extracting cellulose from this promising crop, the constituent composition of the resulting cellulose does not indicate the potential of successfully obtaining spinning solutions. So the goal of this research is to determine the suitability of cellulose samples from miscanthus of the “KAMIS” variety for subsequent spinning solutions and the formation of fibers from them. The resulting fibers’ structural and mechanical properties are determined.

## 2. Materials and Methods

### 2.1. Materials

This study used cellulose samples obtained from Miscanthus giganteus “KAMIS” by the nitric-acid and modified alkaline methods.

The nitric-acid method consists of three steps of treating the feedstock and semi-finished products with diluted solutions at a temperature of 90–95 °C under atmospheric pressure: i—pretreatment of the feedstock with a 1% nitric acid solution to obtain a cellulose-containing product; ii—treatment of the cellulose-containing product with a 4% nitric acid solution to obtain a nitric-acid treatment product; and iii—treatment of the nitric-acid treatment product with a 4% sodium hydroxide solution to obtain cellulose no. I.

The modified alkaline method also has three successive stages of treating the feedstock and semi-finished products with diluted solutions at a temperature of 90–95 °C under atmospheric pressure: j—pretreatment of the feedstock with a 1% solution to obtain a cellulose-containing product; jj—treatment of the cellulose-containing product with a 4% sodium hydroxide solution to obtain a fibrous product; jjj—treatment of the fibrous product with a 4% nitric acid solution to obtain cellulose no. II.

Table 1 shows the constituent composition measurement results: contents (%) of α-cellulose, pentosans, acid-insoluble lignin, ash on an oven-dry basis, cellulose DP, and moisture. The composition of cellulose was determined using widely established “wet” methods [56], whereas the degree of polymerization was determined using cadoxene [56,57].

Both methods allow the obtaining of pure cellulose and are equivalent in terms of reagent consumption, process duration, and energy efficiency. Though having a lower yield, the content of α-cellulose and the cellulose DP in the sample characterize the nitric acid method no. I have higher values than those of cellulose obtained by the modified alkaline method: 88.2 versus 79.7 wt% and 1200 versus 700, respectively. The modified alkaline method guarantees to obtain cellulose with a higher yield, but with a lower cellulose DP.

IR spectroscopy of cellulose samples was carried out using the method in [58]. An X-ray structural analysis (XRD) and the determination of the crystallinity of cellulose were carried out using the method in [58,59].

To obtain cellulose with an average particle size of up to 250 µm, samples were crushed with rollers and then sorted using calibrated sieves.

The solvent employed was N-methylmorpholine-N-oxide (Demochem, Shanghai, China) with a water content of ~10% and a melting point of around 120 °C. Thermooxidative degradation was reduced by adding 0.5% propyl gallate (Sigma-Aldrich, St. Louis, MO, USA) to the solution.

The content of inorganic impurities (metals) in cellulose was determined by the inductively coupled plasma atomic emission spectroscopy (ICP-AES) method (ICPE-9000 by SHIMADZU, Kyoto, Japan). The samples were pretreated with concentrated sulfuric acid, dried on a hotplate under mild conditions, and then burned in a muffle furnace (500 °C). The ash was mineralized in a mixture of concentrated H_2_SO_4_:HNO_3_ acids (1:2) until completely dissolved.

The commercial Lyocell sample was provided by Hyosung Co. (Ulsan, Republic of Korea).

### 2.2. Methods

#### 2.2.1. Preparation of Dopes

The method detailed in [44] was followed to create spinning solutions at several concentrations. Miscanthus cellulose was combined with NMMO and activated in the solid phase. The system was then heated to 120 °C to produce flowable solutions, whose quality was evaluated using optical microscopy (Boetius microscope, VEB Kombinat Nadema, Ruhla, Germany, former GDR).

#### 2.2.2. Rheology

The viscosity and elastic properties of the spinning solutions were evaluated using a HAAKE MARS 60 Rheometer (ThermoFisher Scientific, Dreieich, Germany) (cone-plane geometry, 20 mm diameter and 1° angle) under continuous deformation conditions in the shear rate range γ ˙ from 10^−3^ to 10^3^ s^−1^. The tests were carried out at a temperature of 120 °C. Dynamic tests were carried out in the mode corresponding to the linear viscoelasticity range, in the frequency range (ω) of 0.1–100 Hz at a constant specified stress (τ) of 10 Pa. To avoid contact of the sample with the environment in the gap, the unit was filled with PMS-100 silicone oil (Silan, Moscow, Russia). The tests were carried out in the temperature range of 110–130 °C.

#### 2.2.3. Fibers Spinning

The fibers were spun on a Rheoscope 1000 capillary viscometer (CEAST, Turin, Italy) equipped with a coagulating bath and a fiber winding shaft. The spinning solutions were passed through a capillary with a channel diameter (d) of 0.5 mm and a length (l) of 5 mm, l/d = 10. The air gap between the capillary and the aqueous coagulation bath was 15 cm. The temperature of the coagulation bath was 20 ± 2 °C. The winding speed of the spun fiber reached 100 m/min. Since the path in the coagulation bath was limited, the spun fiber was additionally washed in distilled water until the solvent was completely removed. The washed fiber was dried in a free state at room temperature.

#### 2.2.4. Structure and Morphology

The fiber structure was studied using X-ray diffraction and FTIR spectroscopy. For X-ray diffractometry, we used a Rigaku Rotaflex RU-200 setup equipped with a rotating copper anode (linear focus 0.5 × 10 mm, source operating mode 50 kV/100 mA, wavelength of characteristic CuKα radiation λ = 1.542 Å, secondary graphite monochromator), horizontal goniometer D-Max/B and scintillation detector. X-ray imaging was performed in reflection geometry according to the Bragg–Brentano scheme in the continuous θ–2θ scanning mode in the angular range of 5–45°, at a speed of 2°/min and a scanning step of 0.04°. The measurements were carried out at room temperature. Bundles of at least 100 monofilaments were used as objects. Methods of estimation of crystallinity, crystal size, and background removal are described in French [60], French et al. [61], and Makarov et al. [62].

The IR spectra of the fibers were recorded using a HYPERION-2000 IR microscope and an IFS-66 v/s Bruker IR Fourier spectrometer (crystal–Ge, scan. 50, resolution 2 cm^−1^, range 4000–600 cm^−1^).

The morphology of the surface and transverse cleavages of the fibers was studied by low-voltage scanning electron microscopy (SEM) on a FEI Scios microscope (USA) at an accelerating voltage of less than 1 kV in the secondary electron mode.

#### 2.2.5. Mechanical Testing

Tensile strength, Young’s modulus, and elongation were measured using an Instron 1122 tensile testing machine fitted with pneumatic clamps at a tensile speed of 10 mm/min and a 10 mm gap between the pneumatic clamps in accordance with ISO 6989.

## 3. Results

Miscanthus, like other bast crop, may grow in heavy metal-contaminated soils. During its growth, the plant absorbs significant amounts of lead, iron, and copper, at 6.5, 1.9, and 0.03 g per kilogram of miscanthus biomass, respectively [63]. The process of extracting cellulose from the feedstock is accompanied by a decrease in the content of inorganic substances in the target product. The results of a chemical analysis of metals for the samples studied herein are presented in Table 2.

Regardless of the method of cellulose extraction from miscanthus, the samples contain inorganic components of diverse natures. The use of a modified alkaline extraction method, as opposed to nitric acid, allows one to produce cellulose with a lower metal content. Thus, sample no. I has 5 times more iron than sample no. II. In both cases, the obtained values are more than an order of magnitude greater than those recommended for soluble cellulose (no more than 8 ppm) [40].

**Figure 1:**
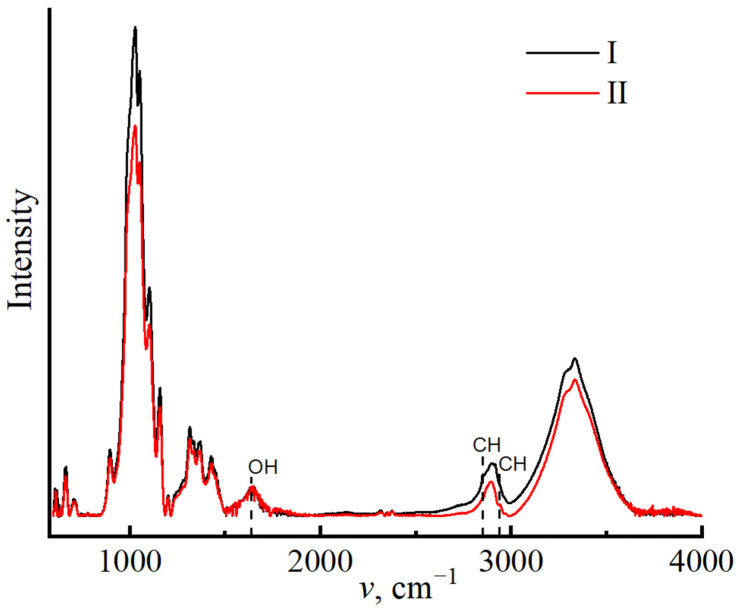
IR spectra of miscanthus cellulose no. I and II.

To confirm the constituent composition of miscanthus cellulose, the IR spectroscopy method was applied, with the results presented in Figure 1.

Regardless of the cellulose isolation method used, the spectrum curves follow a similar path. When comparing the spectra with each other, the following main bands can be distinguished: 898 cm^−1^ (1,4–glycosidic bond); 1107 and 1161 cm^−1^ asymmetric stretching vibrations of the C–O–C bridge, the peaks closer to 1000 cm^−1^ from C–C, C–OH, C–H ring, and side group vibrations; 1315 cm^−1^ (CH2), 1335 cm^−1^ (OH), and 1430 cm^−1^ deformation in-plane vibrations of the OH group; 1620–1641 cm^−1^ (OH) for adsorbed water; and 2850 cm^−1^ (CH), 3100–3600 cm^−1^ (OH). The general appearance of the spectrum suggests that the main part of the sample is represented by native cellulose (polymorph I) [58].

Along with cellulose, bands unique of lignin at 2850 cm^−1^ (C-H) are clearly visible; for sample no. I, the band intensity is much higher than that for sample no. II. The presence of lignin is also indicated by the band at 2942 cm^−1^, corresponding to C-H stretching vibrations in the methyl and methylene groups of lignin [64]. The degree of demethylation and demethoxylation of residual lignin in cellulose samples varies depending on the conditions of cellulose isolation; this is shown in the band’s intensity.

The content of adsorbed moisture in cellulose can be influenced by the non-cellulose components; in the given spectra, the intensity of the band 1637 cm^−1^ for cellulose no. I is higher than that for sample no. II, which corresponds with the data on estimating the amount of equilibrium moisture in cellulose.

IR spectroscopy provides information on structural features, particularly the degree of crystallinity [58]. Cellulose is a polymer with both amorphous and crystalline structures. The ratio of the crystalline and amorphous phases is largely determined by the history of its production. With an increase in the degree of order in the system, the intensity of the regions characterizing the amorphous phase (898 cm^−1^) decreases and increases for the crystalline region (1428–1430 cm^−1^).

The intensity ratio of these bands I_1430_/I_898_ is used to calculate the crystallinity index of cellulose [65]. The calculation of crystallinity indices for miscanthus cellulose revealed differences across samples isolated under various circumstances. The crystallinity index for cellulose no. I is 0.91, whereas sample no. II has lower values of 0.85, indicating more disordered regions in polymer no. II.

**Figure 2:**
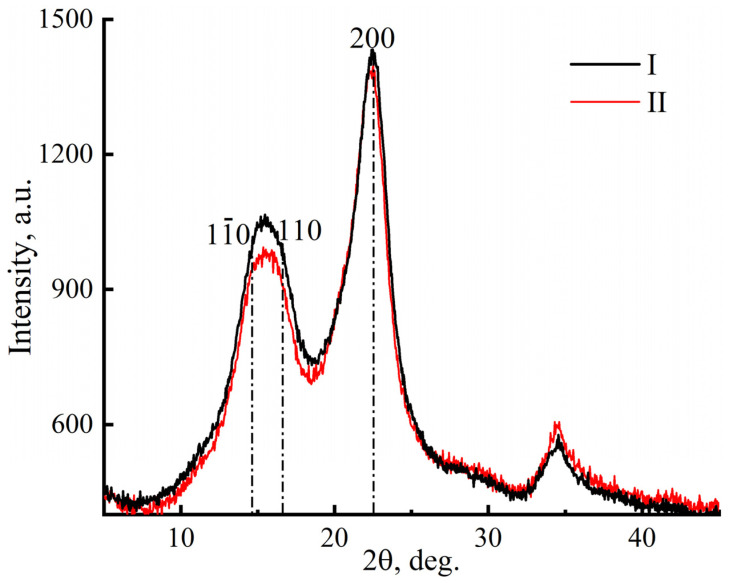
Diffraction patterns of cellulose samples from miscanthus no. I and no. II (reflection mode scanning).

The crystallinity of miscanthus cellulose can also be determined using the X-ray structural analysis method [58] (Figure 2).

The reflection mode is more suitable for determining the degree of crystallinity in cellulose. The diffraction pattern shows three distinct peaks for the crystalline phase of cellulose (1$\bar{1}$0, 110, and 200) at 2θ ~ 14.6°, ~16.6°, and ~22.6° [58], as well as a large halo for the amorphous phase. The detected peak positions characterize native cellulose (polymorph I). The crystallinity of cellulose for samples I and II was 68 and 62%, respectively.

Thus, the cellulose extraction method influences the nature of the change in the constituent composition, degree of polymerization, and supramolecular structural organization. Cellulose no. I contains more residual lignin and, hence, is gray, whereas sample no. II is cream-colored. When spinning solutions are prepared in NMMO, the color difference between the systems is preserved.

**Figure 3:**
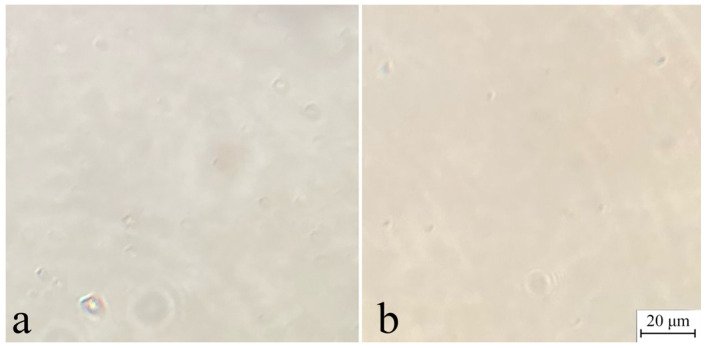
Micrographs of 18% spinning solution of miscanthus cellulose no. I (**a**) and no. II (**b**).

Generally, the resulting solutions are homogenous with little impurities, possibly gels (spots in Figure 3).

**Figure 4:**
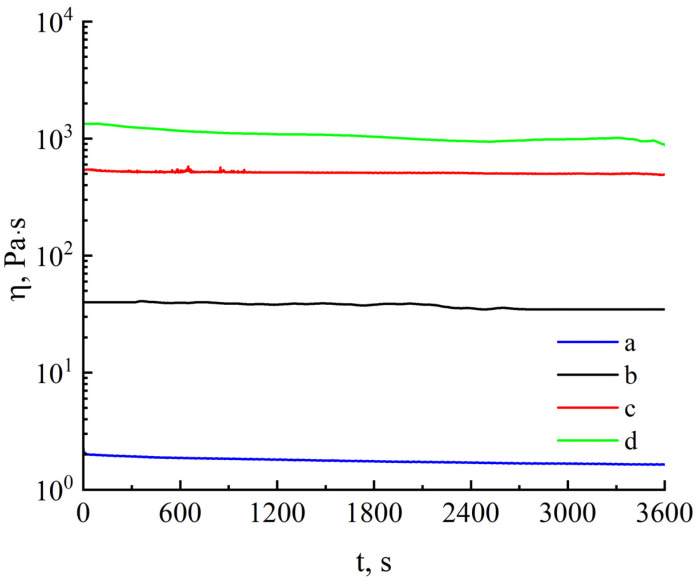
Dependence of viscosity on time for (a) 5, (b) 8, and (c) 16% solutions of cellulose no. I and (d) 18% solution of cellulose no. II in NMMO; T = 120 °C.

The presence of a significant amount of iron in cellulose can negatively affect the quality of spinning solutions. To determine the stability of the solutions, the kinetics of viscosity changes over time was studied, and the results are shown in Figure 4.

The percentage of metals in the system varies with the cellulose content of the solution, which can impact chemical processes in the solution. Figure 4 shows that solutions with a cellulose content of 5 to 18% do not change their viscosity properties within an hour, indicating the system’s stability and the potential of its fiber spinning process over long periods of technological processing.

**Figure 5:**
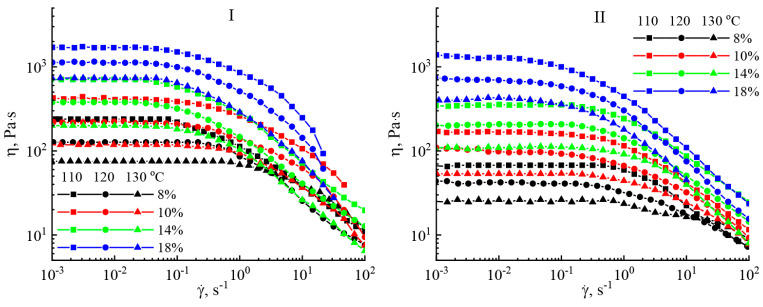
Flow curves of solutions of cellulose samples no. I and II.

Figure 5 shows the flow curves of solutions of miscanthus cellulose samples with a polymer content of 8 to 18% at T = 110 − 130 °C.

The exhibited flow curves are identical independent of the technique of cellulose extraction and its concentration in the solution. On the flow curves, two sections can be distinguished: the first is in the Newtonian region—low shear rates with constant viscosity—where the viscosity starts to drop with increasing shear rates, thus corresponding with the destruction of the internal structure of the solution and the second section being formed. The expected drop in viscosity values is observed as the temperature rises. The area of transition from the Newtonian to structural viscosity moves to the area of lowest shear rates as the content of cellulose in the solution rises. For equiconcentrated solutions under isothermal conditions, the viscosity values for cellulose solutions no. I are higher compared to no. II, which is associated with a higher value of the degree of polymerization of this cellulose.

**Figure 6:**
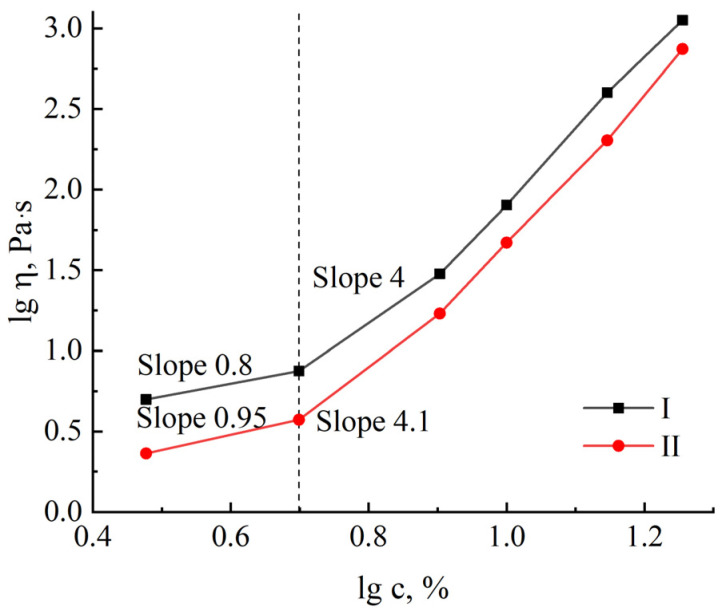
Concentration dependence of viscosity for cellulose solutions no. I and no. II at T = 120 °C ($\overset{\cdot }{\gamma }$ = 0.01 s^−1^).

As clearly observed in Figure 6, a rise in the cellulose content in the system results in a rise in viscosity in line with the power law η = c^α^.

In the concentration dependency graph, two areas stand out that are differentiated by a variable slope of the curve. The first region with a slope tangent value not exceeding 1 refers to the region of dilute solutions, where cellulose macromolecules are swollen globules. High temperatures, at which spinning solutions were achieved, help to induce such a conformation. The slope of the second region rises to 4 ± 1; in such systems, a network of interlocks between macromolecules results [66].

**Figure 7:**
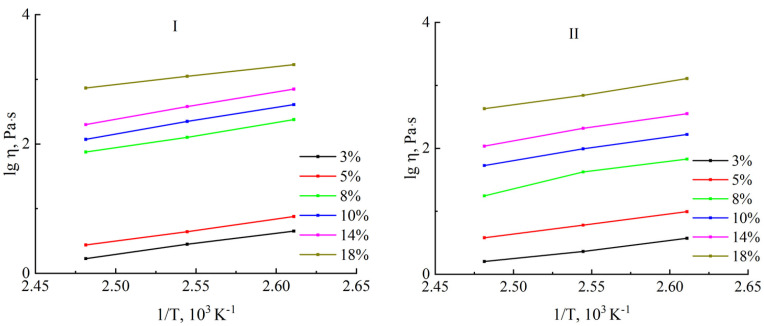
Temperature dependences of the viscosity of solutions of cellulose samples no. I and no. II in the Arrhenius coordinates.

Plotting the logarithm of the initial viscosity as a function of the reciprocal temperature started with the flow curves obtained at various temperatures (Figure 7).

**Figure 8:**
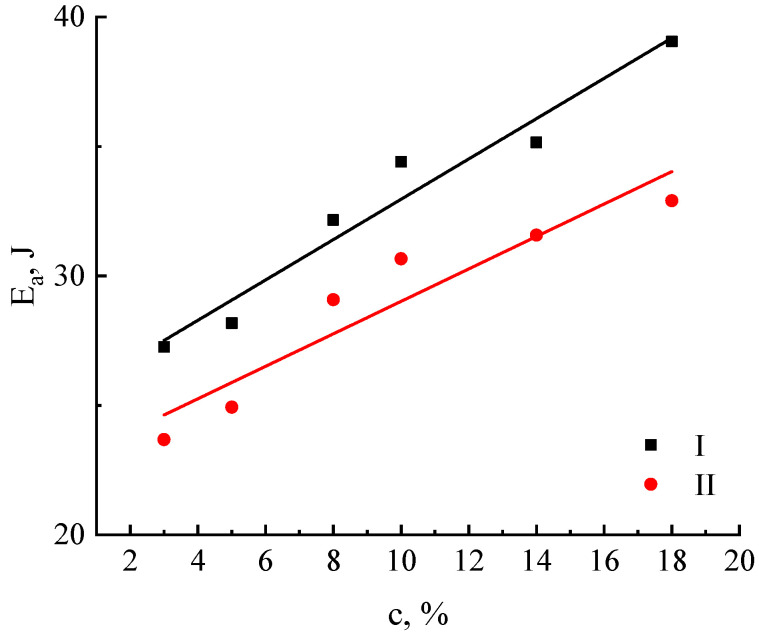
Dependence of the activation energy of miscanthus cellulose solutions no. I and no. II on concentration.

The obtained dependences have a linear form, allowing the estimation of the activation energy of viscous flow *E*. The concentration dependence of the activation energy is shown in Figure 8.

The concentration dependences of the activation energy are somewhat comparable. Being weakly dependent on concentration, the activation energy for cellulose no. I increases monotonically from 27 to 39 kJ/mol and from 24 to 32 kJ/mol for cellulose no. II.

**Figure 9:**
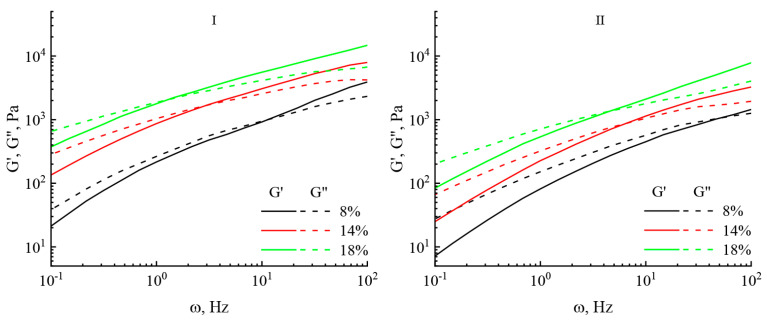
Frequency dependences of the elastic modulus G′ (solid line) and the loss modulus G″ (dashed line) for the studied solutions of miscanthus cellulose no. I and no. II.

Frequency dependences of the dynamic moduli were deduced for all the investigated solutions (Figure 9).

For all samples in the low frequency region, the inequality G″ > G′ is satisfied; i.e., the studied solutions exhibit predominantly viscous properties. With an increase in the frequency of action, the elastic component begins to exceed the viscous one, and the crossover point shifts to the region of lower frequencies. With an increase in the content of both celluloses in the solution from 8 to 18%, both moduli increase by almost an order of magnitude.

**Figure 10:**
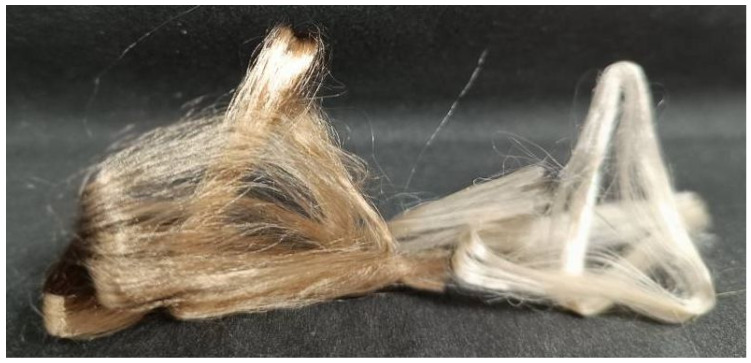
Fibers spun from solutions of miscanthus cellulose no. I (**left** filament bundle) and no. II (**right** filament bundle).

The rheological study data made it possible to choose the ideal concentration and temperature values for fiber spinning. The fibers were spun using a dry-jet wet method. Figure 10 shows photographs of the fibers spun from miscanthus cellulose differing in metal contents.

Sample no. II (right filament bundle) is less colorful and has a 79.7% alpha fraction. Brown fiber made from cellulose no. I has an alpha fraction of 88.2%. A greater proportion of residual lignin, which typically contains metals such as iron, is most likely responsible for the brown color. Despite their differing hues, both samples have a glossy appearance and are smooth to the touch, which is particularly appealing for Lyocell fiber.

**Figure 11:**
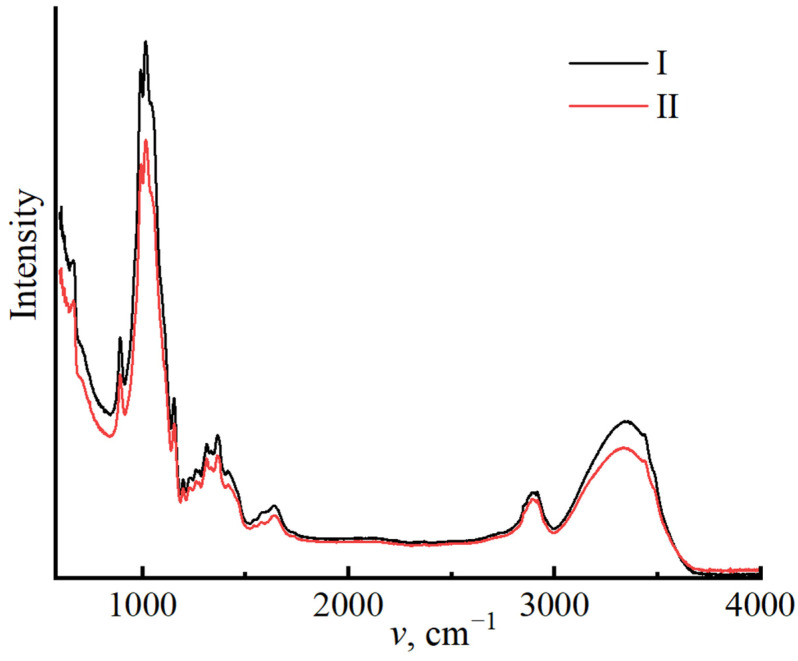
IR spectra of fibers spun from cellulose no. I and no. II.

**Figure 12:**
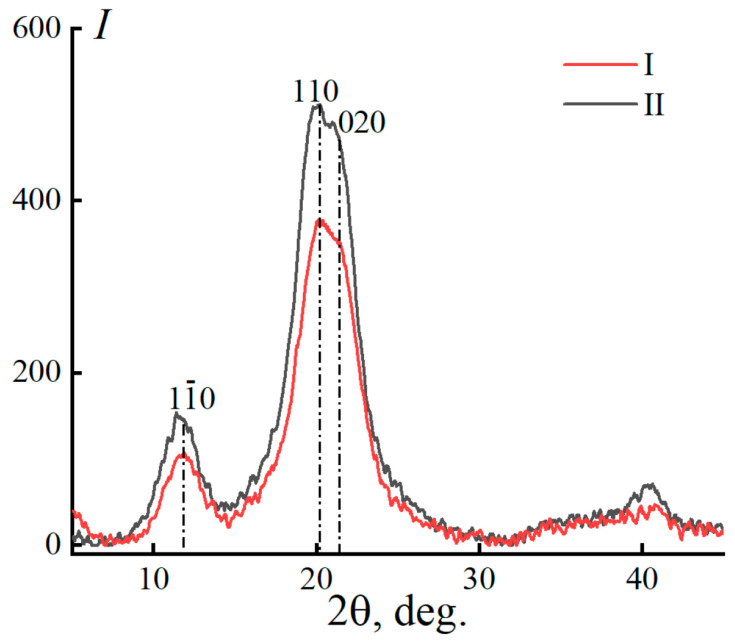
Equatorial diffraction patterns of fibers spun from 18% solutions of miscanthus cellulose no. I and no. II in NMMO.

The structure of cellulose is directly affected by its dissolution and regeneration in the form of fibers, as demonstrated by IR spectroscopy (Figure 11) and X-ray diffraction (Figure 12).

The structure of regenerated cellulose, like that of original cellulose, is dictated by its hydroxyl groups participating in the formation of the amorphous and crystalline regions. To analyze the transformation of the cellulose structure, O’Connor proposed using the spectral region of 1500–850 cm^−1^ [67]. After regeneration, the cellulose I bands shift or vary in intensity. A decrease in the intensity of the bands for regenerated cellulose is also noted in the region of 3600–3100 cm^−1^. In the presented spectrum, the bands at 3439 and 3342 cm^−1^ for regenerated cellulose II are most clearly visible [68]. Crystallinity indices for samples no. I and no. II were determined from the amorphous and crystalline bands in regenerated cellulose, resulting in 0.35 and 0.34, respectively.

The regeneration of solutions in water leads to a change in the diffraction pattern of cellulose (Figure 12).

**Figure 13:**
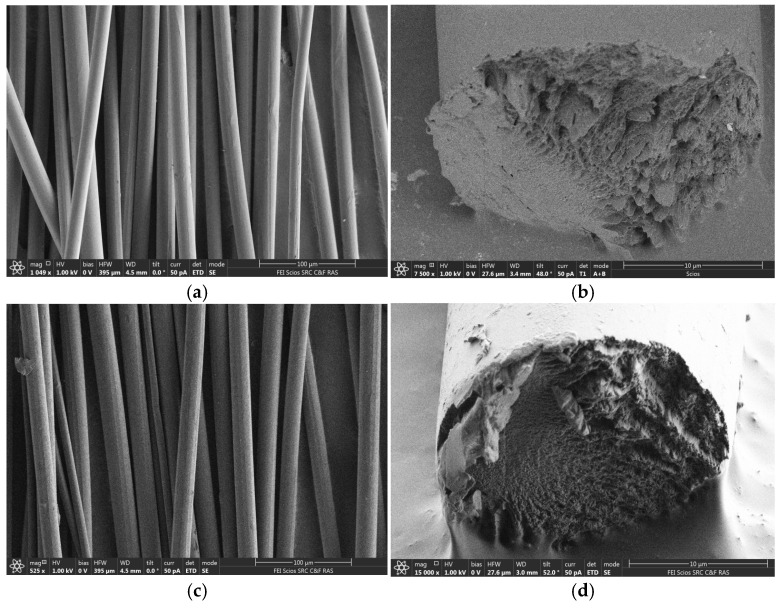
SEM images of the surface and cleavages of fibers (**a**,**b**) no. I and (**c**,**d**) no. II at different zoom.

A comparison of Figure 12 and Figure 2 reveals that the initial peak of native cellulose shifts to a lower angle range of 2θ ~ 12.1° (plane (1 $\bar{1}$0)). The peak corresponding to the plane (110), on the contrary, shifts to larger angles, in the region of 2θ ~ 20.1°. The third peak is in the region of 2θ ~ 20.1° (plane 020). The shift in the position of cellulose’s main peaks corresponds to a structural alteration, specifically the move from a parallel arrangement of macromolecules to an antiparallel one (polymorph II) [68,69,70]. The degree of crystallinity values obtained for the fibers are far smaller than those of the data acquired for the original cellulose. For fibers no. I, the degree of crystallinity is slightly higher compared to no. II and is 52 and 48%, respectively. Figure 13 depicts the microstructure of fibers spun from miscanthus cellulose.

It is well recognized that unlike bast crops like flax, hemp, and cotton [71], isolating natural fibers from miscanthus without chemical treatment processes is entirely impossible because it is a cereal crop. However, the authors of [72] depict the morphology of fibers isolated from miscanthus seeds. Unlike natural miscanthus fibers (cellulose content 67%), the length of which varies from 8 to 12 mm, artificial fibers based on its cellulose have an “unlimited” length. The diameters of the spun fibers, like natural ones, have an insignificant deviation from the average value, namely 15 and 9 μm, respectively. Natural fibers have an uneven surface with cracks and grooves, as well as locally deposited wax. The surface of the produced fibers is unusually smooth, with no clearly visible defects; on the surface, one can see re-deposited low-molecular components in the form of build-ups and particles. Miscanthus fibers, both manufactured and natural, have a nearly spherical cross-section. Natural fibers have hollow cores, whereas spun fibers are homogenous and monolithic.

Textile fibers must possess high strength and deformation characteristics. The inclusion of contaminants (non-cellulose components and metals) in the system can reduce the mechanical characteristics of the materials. Table 3 compares the mechanical characteristics of regenerated fibers from miscanthus cellulose with varying levels of contaminants to the industrial sample Lyocell.

The strength of polymer fibers is determined by the degree of polymerization and macromolecule orientation during the spinning process. Cellulose no. I fibers with higher polymerization levels exhibited the highest strength. The elastic moduli are also higher for fibers no. I compared to samples no. II and the commercial Lyocell fiber spun from wood cellulose. The elongation at break for the miscanthus cellulose fiber is higher than that of commercial Lyocell. Consequently, the mechanical behavior of miscanthus cellulose fibers with a considerable impurity concentration matches that of industrial cellulose fibers.

## 4. Conclusions

Being a perennial plant, miscanthus can be used as the raw material base to produce cellulose. Its ability to sorb inorganic compounds in large quantities is used for soil phytoremediation. As a result, to obtain high-quality soluble cellulose, it is recommended to employ soils with lower metal concentrations, etc. The method used to isolate cellulose from Miscanthus var. “KAMIS” can be an important aspect in ensuring the constituent composition of cellulose, particularly in terms of non-cellulosics and inorganic contaminants. It is demonstrated that the modified alkaline method of isolation yields cellulose with a lower DP and a lower metal concentration than the nitric-acid method. Despite the higher iron content, both celluloses are well soluble in NMMO to give spinning dopes with concentrations of up to 18%. Cellulose solutions in NMMO are relatively stable at 120 °C. According to rheological characteristics, 16% solutions were chosen as the most suitable for spinning, from which fibers were successfully spun. The presence of residual lignin and iron determines the color of the resulting fiber. The mechanical properties of cellulose fibers are determined by cellulose’s structural ordering and DP. The nitric-acid process produces cellulose fibers with the highest strength and elastic modulus values. In contrast, the fibers spun from cellulose generated by the modified alkaline technique have higher deformation properties. The comparison between the mechanical properties of miscanthus fibers and the commercial Lyocell sample made of wood cellulose revealed their similarity.

## Figures and Tables

**Table 1:** Results of determining the constituent composition and DP of cellulose samples no. I and II.

Characteristics	Cellulose No. I	Cellulose No. II
α-cellulose, wt%	88.2 ± 0.5	79.7 ± 0.5
Pentosans, wt%	1.6 ± 0.1	1.9 ± 0.1
Acid-insoluble lignin, wt%	0.80 ± 0.05	0.30 ± 0.05
Ash, %	0.40 ± 0.01	0.1 ± 0.01
Degree of polymerization	1200 ± 100	700 ± 50
Moisture, %	6.7	5.0

**Table 2:** Metal contents in cellulose samples no. I and II.

	Metal Contents, ppm	
	Cellulose No. I	Cellulose No. II
Al	47.66	25.98
Ba	2.54	0.72
Ca	1050.92	871.78
Cr	25.34	3.73
Cu	6.86	16.53
Fe	520.45	111.81
Li	7.34	≤0.02
Mg	87.47	103.22
Mn	4.22	0.08
Na	1666.38	1837.70
Ni	23.04	6.06
Sn	28.80	30.88
Zn	20.23	3.39

**Table 3:** Mechanical properties of regenerated fibers from miscanthus cellulose in comparison with the industrial sample Lyocell.

Fiber	Diameter, µm	Tensile Strength, MPa	Elastic Modulus, GPa	Elongation at Break, %
no. I	23–27	420–580	14.0–19.0	6.3–11.2
no. II	12–29	360–490	11.0–16.5	8.2–14.5
Commercial Lyocell	18	610	14	7

## Data Availability

Data is contained within the article.
